# Cardiac vein retroinjections provide an efficient approach for global left ventricular gene transfer with adenovirus and adeno-associated virus

**DOI:** 10.1038/s41598-024-51712-5

**Published:** 2024-01-17

**Authors:** Jaakko Lampela, Juho Pajula, Niko Järveläinen, Satu Siimes, Nihay Laham-Karam, Antti Kivelä, Isidore Mushimiyimana, Jussi Nurro, Juha Hartikainen, Seppo Ylä-Herttuala

**Affiliations:** 1 A.I.Virtanen Institute for Molecular Sciences, University of Eastern Finland, Kuopio, Finland; 2 Heart Center, Kuopio University Hospital, Kuopio, Finland; 3 Gene Therapy Unit, Kuopio University Hospital, Kuopio, Finland; 4 Heart Hospital, Tampere University Hospital, Tampere, Finland

**Keywords:** Interventional cardiology, Cardiovascular diseases, Cardiomyopathies, Heart failure, Vascular diseases, Gene therapy, Targeted gene repair, Therapeutics, Preclinical research, Translational research, Cardiology, Health care, Medical research, Molecular medicine

## Abstract

Heart failure (HF) is a major burden worldwide, and new therapies are urgently needed. Gene therapy is a promising new approach to treat myocardial diseases. However, current cardiac gene delivery methods for producing global myocardial effects have been inefficient. The aim of this study was to develop an endovascular, reproducible, and clinically applicable gene transfer method for global left ventricular (LV) transduction. Domestic pigs (n = 52) were used for the experiments. Global LV myocardium coverage was achieved by three retrograde injections into the three main LV vein branches. The distribution outcome was significantly improved by simultaneous transient occlusions of the corresponding coronary arteries and the main anastomotic veins of the retroinjected veins. The achieved cardiac distribution was visualized first by administering Indian Ink solution. Secondly, AdLacZ (2 × 10^12^vp) and AAV2-GFP (2 × 10^13^vg) gene transfers were performed to study gene transduction efficacy of the method. By retrograde injections with simultaneous coronary arterial occlusions, both adenovirus (Ad) and adeno-associated virus (AAV) vectors were shown to deliver an efficient transduction of the LV. We conclude that retrograde injections into the three main LV veins is a potential new approach for a global LV gene transfer.

## Introduction

Myocardial diseases and heart failure (HF) are a major health burden globally^1^. Gene therapy is an attractive approach to treating cardiac diseases since it can provide targeted and long-lasting effects by single dosing. The main challenge in cardiac gene therapy has been an inefficient delivery of vectors to the myocardium^2^.

Endomyocardial injections^2,3^ and pressure-regulated coronary venous infusion^4^ are the most efficient for achieving myocardial cell transduction among the current percutaneous cardiac gene delivery methods. However, endomyocardial injections provide only spatially very targeted transgene expression, whereas pressure-regulated coronary infusion produces transgene expression only in the territory of the left anterior descending coronary artery (LAD)^5^. Intracoronary and intrapericardial delivery are global methods but limited in efficacy in transducing heart muscle ^6–8^. Recirculating vectors, however, can improve the efficacy of coronary infusion^9^.

Adenovirus (Ad) is a frequently used viral vector, and has been shown to be safe in clinical trials when administered locally^10–13^. Ad-mediated transgene expression lasts approximately two weeks after the gene transfer^14^. Instead, adeno-associated virus (AAV) produces long-term transgene expression^15^. Various AAV serotypes differ significantly in immune response, tropism, and transduction efficacy in different tissues^15–17^.

Pig was chosen as an experimental animal because of the high cardiac anatomical and physiological similarity to human^18^.

The aim of this study was to develop a feasible, reproducible, and efficient method for viral vector mediated global left ventricular (LV) gene transfer. Guided by the results of published cardiac delivery methods, retrograde approach via cardiac veins was chosen as the basis of the method development.

## Methods

### Animal model

The animal experiments were approved by National Animal Experiment Board in Finland. Healthy female Finnish landrace pigs weighing 30–45 kg at the start of the experiment were used in the study (n = 52).

### Study overview

At first, the distribution patterns of the retroinjections were assessed by injecting Indian Ink (Royal Talens, Netherlands) retrogradely into the cardiac veins. Experiments were then performed with Ad and AAV vectors to confirm feasibility of the method for cardiac gene transfer. Prior to the experimental setups described below, fifteen pigs were used to develop a reliable catheterization protocol ensuring access to porcine coronary vein branches.

*In the first set of experiments*, we examined the effect of LAD occlusion during the retroinjection into the anterior cardiac vein (ACV). Indian ink and AdLacZ (dose 4.8 × 10^12^ vp) were injected into ACV, with or without LAD occlusion. In total, four pigs received Indian ink, and two pigs received AdLacZ.

*In the second set of experiments*, the ability to achieve widespread distribution in LV was examined by ink injections into the three main LV veins (ACV, left marginal vein, LMV, or left posterior marginal vein, LPMV and middle cardiac vein, MCV) with simultaneous occlusions of the corresponding arteries (LAD, left circumflex artery, LCx, and right coronary artery, RCA), respectively, and the injection volumes to each vessel pair were defined. Procedures to each vessel pair were carried out in different animals to see the specific distribution outcome of each retroinjection. In total, fifteen pigs were included in this set of experiments.

*In the third set of experiments*, retroinjections into the three main cardiac veins with AdLacZ (total dose 2 × 10^12^ vp) and AAV2-GFP (total dose 2 × 10^13^ vg) with simultaneous occlusions of the corresponding arteries were performed. Since the difference in the transgene expression kinetics of the vectors, a subgroup (n = 2) of the Ad animals was sacrificed at six days, and another subgroup of the Ad animals (n = 3) and all AAV2 (n = 3) animals were sacrificed one month after the gene transfer. Vector distribution in the heart and non-target tissues was analyzed by PCR. Histology and general pathology of the main internal organs were also studied.

*In the fourth set of experiments,* the effect of occlusion of the main venous anastomosis was studied. Also, we studied the outcome in LV anterior wall (LVAW) when half a dose was administered via a microcatheter into the apical part of ACV and the remaining half of the dose via the occlusion balloon into the proximal ACV. In total, eight animals were included in this set of experiments.

### Catheterization protocol

#### Coronary angiogram and coronary artery cannulation

A 6F introducer sheath was placed in the femoral artery, and the coronary arteries were selectively cannulated by a standard 6F guiding catheter. To determine individual variations in vascularity, coronary arteries and veins were imaged by antegrade injections of contrast agent. Using a standard 0.014 guidewire as support, an over-the-wire (OTW) coronary balloon catheter was advanced into the proximal coronary artery.

#### Cardiac vein cannulation

An 8F introducer sheath was placed in the femoral vein and a shortened 7F guiding catheter with large inner diameter (Launcher, Medtronic) was advanced to the ostium of the great cardiac vein, the most prominent vein of the LV lateral wall (LVLW) or the MCV. With the support of the guiding catheter and a hydrophilic 0.032″, 0.025″ or 0.018″ guidewire, a 4-6F wedge pressure balloon catheter was advanced selectively one by one into the three main veins of the LV for the retroinjection procedure.

In the fourth set of experiments, a 2.5F microcatheter was inserted into the apical part of the ACV through the 6F wedge pressure balloon catheter, using the standard 0.014″ guidewire. For occluding the main venous anastomosis, a 4-5F wedge pressure balloon catheter was inserted into the main anastomotic vein of the target vein via a 7F guiding catheter (Launcer, Medtronic) and an 8F introducer sheath placed in the external jugular vein.

#### Retroinjection procedure

When the OTW balloon catheter was in the target coronary artery and the wedge pressure balloon catheter in the regionally corresponding vein, the artery was occluded by inflating the OTW balloon. Immediately after the balloon inflation, 5 ml of 0.9% NaCl solution was administered through the OTW balloon catheter to reduce the amount of blood in the region. Next, the wedge pressure balloon in the regional cardiac vein was inflated, and the ink solution or the viral vectors were retroinfused through the balloon within 1 min, starting from the inflation of the OTW balloon in the coronary artery. After one minute, the OTW balloon was deflated and withdrawn into the guiding catheter. After further 9 min, the wedge pressure balloon was deflated and removed from the vein.

In the fourth set of experiments, the wedge pressure balloon in the main anastomotic vein was inflated just before the inflation of the wedge pressure balloon in the target vein. The anastomotic vein was released immediately after the retroinjection. To study the effect of using the microcatheter, half of the ink solution was administered via the microcatheter into the apical ACV, and the rest into the basal ACV through the wedge pressure balloon catheter, immediately after the balloon inflations.

**Figure 1:**
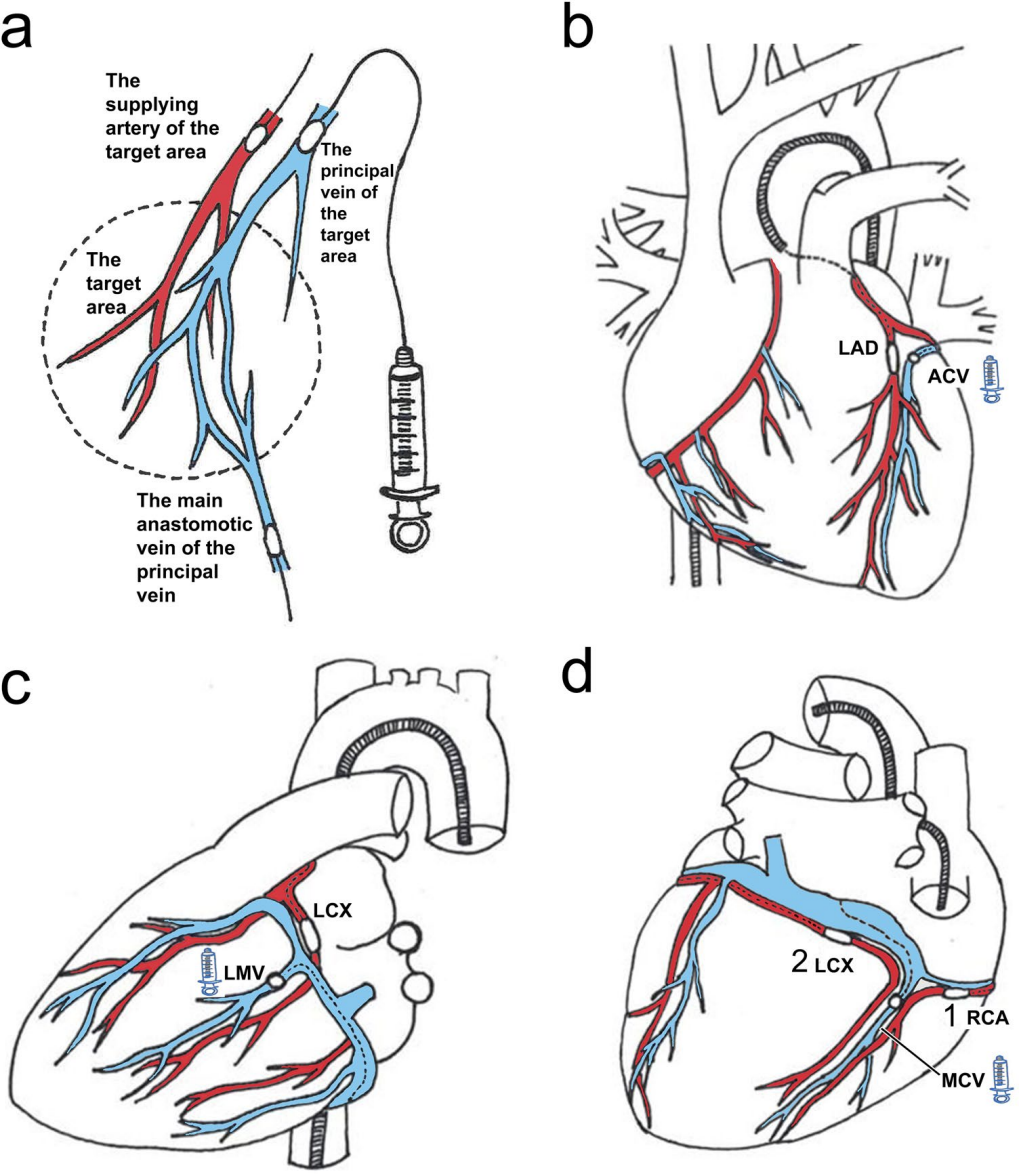
Catheterization setup. (**A**) Schematic about the catheterization setup of the method. The retroinjection was given through the wedge pressure balloon catheter placed in the principal vein of the target area, during simultaneous occlusion of the supplying coronary artery of the target area. Also, the main anastomotic vein of the target area can be occluded. (**B**) To treat the LVAW and anterior IVS, retroinjection was given into the ACV, during simultaneous transient LAD occlusion. (**C**) To treat the LVLW, retroinjection was given into the most prominent vein of the region (LMV or LPMV), and LCx was occluded transiently during the retroinjection. (**D**) To treat the LVPW and posterior IVS, retroinjection was given to the MCV. During retroinjection,either RCA (1.) or LCx (2.), depending of which one is dominant in posterior LV, was occluded. (**B**,**C**,**D**) In some animals, the main anastomotic vein of each retroinjected vein was occluded during the retroinjections. (**B**,**C**,**D**) The vessel of retroinjection is marked with a syringe symbol.

The catheterization setup of the procedure is presented in Fig. 1.

### Vector dilution and dosing

Of the total dose of the vector or the ink solution, 50% was administered into ACV, 25% into MCV and 25% into the most prominent vein of the LVLW: either LMV or LPMV. Viral vectors were diluted with 0.9% NaCl solution. In the ink experiments, 20 ml of the ink solution was administered into ACV, 10 ml into MCV, and 10 ml into the most prominent lateral vein. In the gene transfer experiments, 15 ml of the vector solution was administered into ACV and 7.5 ml into both MCV and LMV/LPMV. Ad5 vector coding for nuclear targeted β-galactosidase and AAV2 vector coding for GFP were produced in the National Virus Vector Laboratory (A.I.Virtanen Institute, Biocenter Kuopio) as described^11,19^

### Anesthesia and medication

Animals were sedated with intramuscular administration of xylazine 0.2 ml/kg and midazolam 0.2 ml/kg. General anesthesia was achieved with propofol with individually adjusted infusion rate and fentanyl 10 ug/kg/h i.v. Animals were intubated and mechanically ventilated. For postoperative analgesia, buprenorphine 0.3 mg i.m. or/and carprofen 4 mg/kg i.m. were given.

Before the invasive procedures, pigs received prophylactic cefuroxime 500 mg i.m. To prevent arrhythmias during catheterization, animals were given 100 mg of lidocaine i.v. and 615 mg of MgSO_4_ i.v. 30 mg of enoxaparin i.v and isosorbide dinitrate s.l. were administered after the insertion of the introducer sheaths. At the end of the experiment, animals were sacrificed with saturated potassium chloride solution i.v, after a terminal load of propofol.

### Samples

Blood samples were taken just before the gene transfer and at sacrifice. Day six blood samples were also collected from Ad animals. Serum was isolated and stored in − 80 °C. Troponin I (TnI), creatinine (crea), lactate dehydrogenase (LDH), C-reactive protein (CRP), alkaline phosphatase (ALP), and alanine aminotransferase (ALAT), were measured from serum by veterinary clinical chemistry laboratory (Movet Oy, Kuopio).

**Figure 2:**
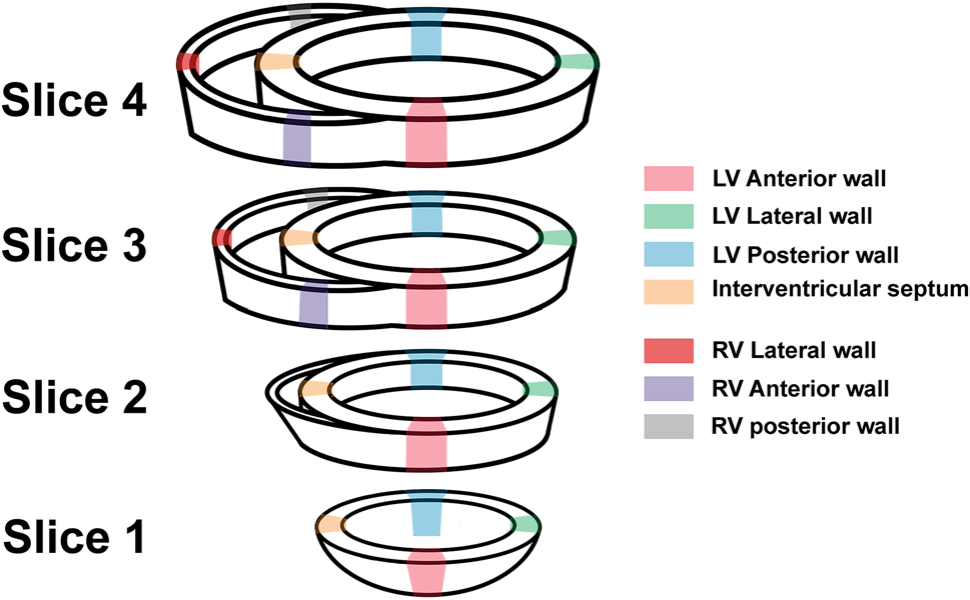
Sampling sites of the cardiac ventriculi. Of each site, both histological and snap-frozen samples were collected.

After sacrifice, the heart was removed, cut horizontally into four slices and photographed. The hearts of animals receiving the vector solutions were perfused with PBS before slicing. LV samples were collected from LVAW, LVLW, LV posterior wall (LVPW) and interventricular septum (IVS). To determine the biodistribution outside of the LV, samples were collected from the anterior, lateral, and posterior wall of the right ventricle (RVAW, RVLW, and RVPW, respectively), left and right cardiac atrium (LA and RA), lung, liver, spleen, kidney, ovary, and lymph nodes from para-aortal site and groin. Sampling sites of the cardiac ventriculi are presented in Fig. 2.

Part of the tissue samples were immediately snap-frozen in liquid nitrogen and stored in − 80 °C for PCR (polymerase chain reaction) analysis. Rest of the tissue samples were immersion fixed for 48 h in 4% paraformaldehyde and further in 15% sucrose, before embedding in paraffin^20^. Polyclonal rabbit IgG antibody (A-11132, Invitrogen) was used to stain β-galactosidase positive nuclei in the AdLacZ transduced hearts^20^.

### Biodistribution analysis

Both AAV and Ad vector genomes in all sampling sites were quantified by qPCR. Tissue DNA was extracted using NucleoSpin® DNA RapidLyse kit (Machery-Nagel), according to the manufacturer’s instructions. The total amount of vector was quantitated using AAV2 ITR or LacZ specific PrimeTime® qPCR assay (Integrated DNA Technologies) and TaqMan Fast Advanced Master Mix (Applied Biosystems) and measured in StepOnePlus™ Real-Time PCR instrument (Applied Biosystems)^19^

### Transgene expression analysis

Both AAV and Ad mediated transgene expression in all samples was quantified by qRT-PCR. Tissue RNA was extracted using TRI Reagent® (Life Technologies) and treated with DNAse (DNA-Free™, Life Technologies), according to the manufacturer’s instructions. cDNA was synthesized from 1 µg of total RNA using RevertAid Reverse Transcriptase (Thermo Scientific) and Random Hexamer Primers (Thermo Scientific). The levels of GFP and LacZ cDNA were measured by qPCR using GFP or LacZ specific Taqman-based assays (Integrated DNA Technologies), and normalized to the expression of the housekeeping gene HPRT (Applied Biosystems; ss03388274 m1 HPRT1)^19^

### Statistics

Vector genome distribution results are expressed separately for each individual animal in dot plots, the line indicating the average value for the group. Statistical significance was evaluated by one-way ANOVA with Bonferroni’s post hoc test or paired t-test as appropriate. A value of p < 0.05 was considered statistically significant. Computations were performed with Prism 5 version 5.03 (GraphPad Software, California, USA).

### Approval for animal experiments

All animal experiments were approved by the Animal Experiment Board in Finland and carried out in accordance with The Finnish Act on Animal Experimentation. The investigation conforms with the Guide for The Care and Use of Laboratory Animals published by the US National Institutes of Health (NIH Publication No. 85-23, revised 1996). The ARRIVE guidelines were compiled in all the used methods.

## Results

**Figure 3:**
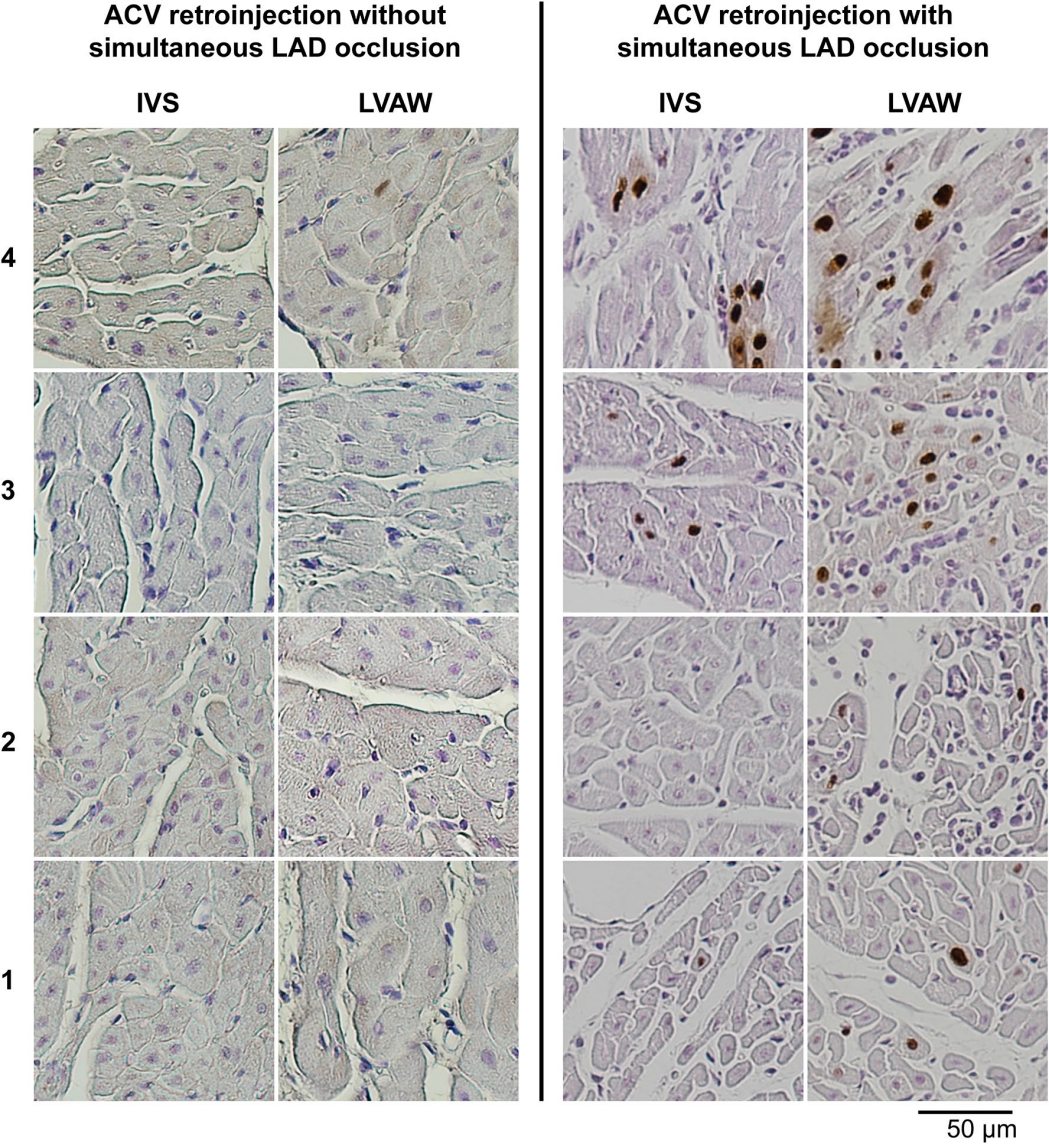
Beta-galactosidase immunostainings of LVAW and IVS from animals of the first set of experiments. The samples on the left are from the animal that underwent ACV retroinjection without LAD occlusion. On the right are samples from the animal that underwent ACV retroinjection with transient LAD occlusion. The sections are photographed at 20 × magnification. Samples were collected from the LVAW and the IVS as presented in Fig. 2.

In the first set of experiments, much wider distribution of the injected ink was obtained by simultaneous LAD occlusion during the retroinjection into the ACV. Similar findings were obtained from the AdLacZ gene transfer experiments: When ACV retroinjection was administered without LAD occlusion, individual LacZ-positive cells were detected only in the basal part of LVAW. In contrast, when transient LAD occlusion was used, LacZ-positive cells were found throughout the LVAW and the IVS (Fig. 3). The other sites of sampling indicated no signals of transduction.

**Figure 4:**
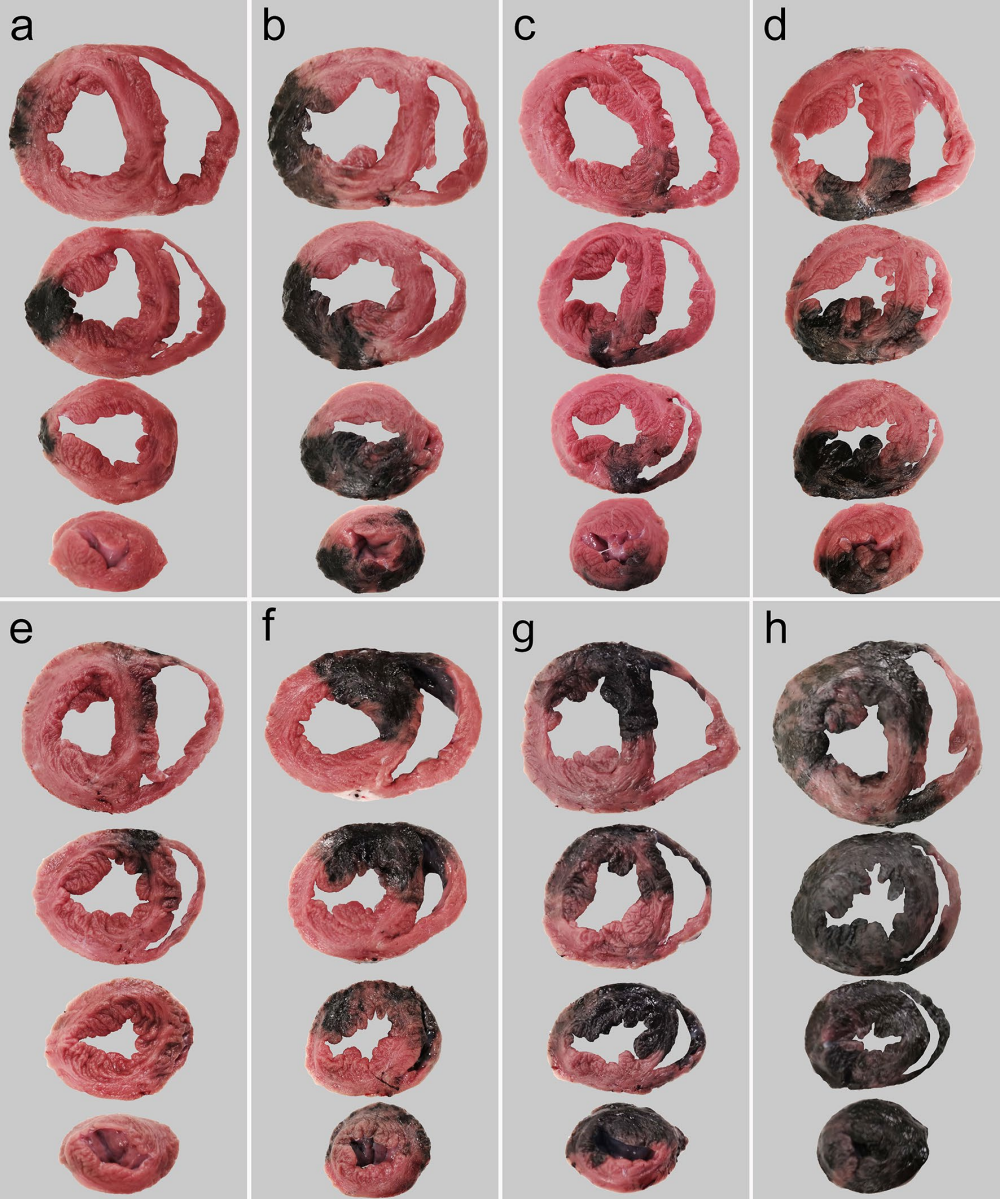
Ink distribution after different retroinjection procedures. The heart slices are oriented as anterior wall upwards. (**A**) 10 ml retroinjection into the LMV with a simultaneous LCx occlusion. (**B**) 10 ml retroinjection into the LMV with simultaneous LCx and MCV occlusions. (**C**) 10 ml retroinjection into the MCV with a simultaneous RCA occlusion. (**D**) 10 ml retroinjection into the MCV with simultaneous RCA and ACV occlusions. (**E**) 20 ml retroinjection into the ACV with a simultaneous LAD occlusion. (**F**) 20 ml retroinjection into the ACV with simultaneous LAD and MCV occlusions. (**G**) 20 ml retroinjection into the ACV with simultaneous LAD and MCV occlusions. Half of the ink solution was administered through a microcatheter placed in the apical part of the ACV. (**H**) Distribution outcome of retroinjections into the three main LV veins, with simultaneous transient occlusions of the coronary arteries and the main anastomotic branches of the cardiac veins. The retroinjections were performed as described in the Fig. 1.

In the second set of experiments, retroinjection into the LMV with a simultaneous LCx occlusion resulted in a wide colouration of the LVLW (Fig. 4a). Similarly, during the transient occlusion of the dominant coronary artery of the LVPW, a retroinjection into the MCV resulted in a wide ink distribution in the LVPW and posterior part of the IVS (Fig. 4c).

**Figure 5:**
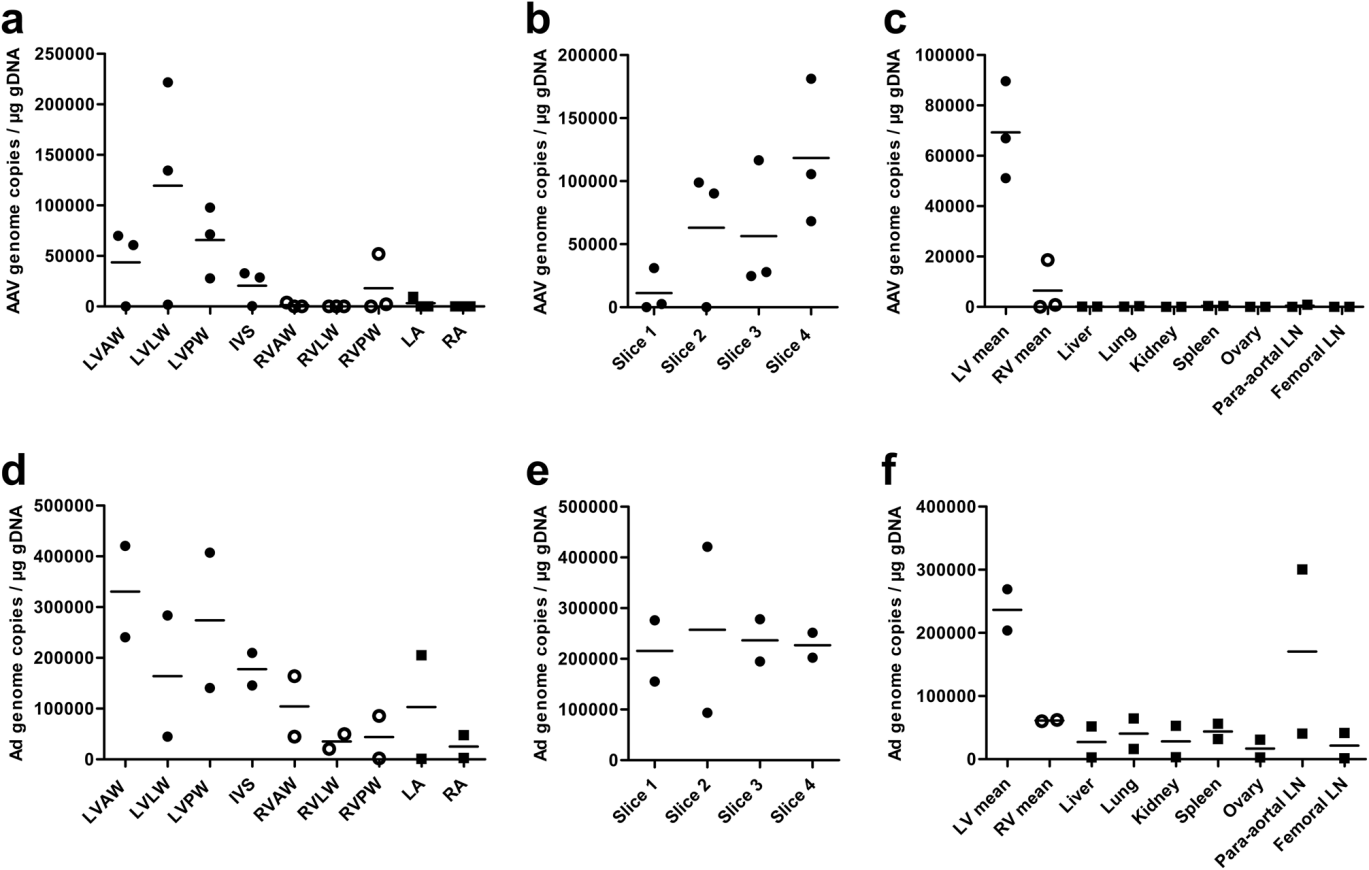
Vector genome distribution in heart and extracardiac tissues. Figures A-C are from AAV2-GFP animals at 1 month timepoint. Figures D-F are from AdLacZ animals at six-day timepoint. (**A**) AAV genome intracardiac distribution. (**B**) Vertical LV distribution of AAV genome. (**C**) AAV genome biodistribution. (**D**) Ad genome intracardiac distribution. (**E**) Vertical LV distribution of Ad genome. (**F**) Ad genome biodistribution. LV mean includes and RV mean excludes the IVS. Each dot indicates one animal’s mean value of the collected samples from the area in question. LN indicates lymph node. Cardiac sampling sites are presented in Fig. 2. Closed circles: LV; open circles: RV.

In the third set of experiments, the delivery method without either the anastomotic vein occlusions or the microcatheter was tested by gene transfers with AdLacZ and AAV2-GFP. Vector genome distribution in the heart and extracardiac tissues quantified by qPCR are shown in Fig. 5*.* Transgene expression quantifications by mRNA qRT-PCR analysis are presented in Table 1.

**Table 1:** Transgenic mRNA expression in cardiac and extracardiac samples. Results are presented as quantity of the transgenic mRNA divided by the quantity of the HPRT. AAV-GFP animals were analyzed at one month timepoint, and AdLacZ animals at six-day timepoint. LV mean includes and RV mean excludes the IVS. Results are presented as mean ± SEM of the collected samples from the area in question. LN = lymph node. ND = not detected. Cardiac sampling sites are presented in Fig. 2.

Group, timepoint	Transgenic mRNA/HPRT	
	AAV2-GFP, one month	AdLacZ, six days
LVAW mean	328.1 ± 83.5	72.0 ± 5,25
LVLW mean	4176.0 ± 4166.3	21.7 ± 4,95
LVPW mean	2423.3 ± 1557.9	1890.5 ± 1884,4
IVS mean	146.1 ± 39.3	901.1 ± 893,7
Slice 1 mean	804.9 ± 748.9	2443.2 ± 2442,1
Slice 2 mean	1711.65 ± 868.0	4.5 ± 2,95
Slice 3 mean	948.9 ± 771.9	288.4 ± 257,75
Slice 4 mean	3608.1 ± 3176.6	149.2 ± 70,9
LV mean	1768.4 ± 1400.4	721.3 ± 692
RVAW mean	ND	33.0 ± 19.95
RVLW mean	ND	1.15 ± 1.15
RVPW mean	2441,25 ± 2314,45	1.15 ± 1,15
LA	ND	ND
RA	ND	36.2 ± 36.2
Lung	ND	0.5 ± 0.5
Liver	ND	ND
Spleen	ND	ND
Kidney	ND	1.5 ± 1.5
Ovary	ND	0.8 ± 0.8
Para-aortal LN	ND	1.0 ± 1.0
Femoral LN	ND	ND

Finally, the fourth set of experiments revealed even a significantly wider distribution of ink by adding a transient occlusion of the main anastomotic veins of each vein to be retroinjected (Fig. 4b,d,f). In addition, administering half of the dose to the distal ACV via a microcatheter provided a better outcome in the apical myocardium (Fig. 4g).

AAV2 genome and GFP mRNA were most strongly present in basal parts of the LV, but there was also evident positivity in the apical sites (Fig. 4, Fig. 5b, Table 1). Also, RV sites near the IVS were positive, congruently with the ink experiments (Fig. 5a, Table 1). Only very low levels of AAV2 genome and no mRNA encoding GFP were detected in the extracardiac tissues (Fig. 5a, Table 1).

Ad genome was more evenly present in the LV, when compared to the AAV2 distribution (Fig. 5b,e). Small amounts of Ad genome were detected in the extracardiac samples (Fig. 5f). From extracardiac tissues, transgenic LacZ encoding mRNA was detected in the lung, kidney, ovary, and para-aortal lymph node (Table 1).

Assuming a mean of 12.3 pq of DNA content per heart cell^21^ and thus ⁓81,300 cells per µg DNA, the mean amount of obtained copy number in LV was ⁓85% of the number of cells in AAV transduced animals. Instead, in Ad transduced animals, the average amount of vector genome copies in the LV was even higher, indicating high transduction efficiency (Fig. 5).

Histological assessment with hematoxylin–eosin-stained sections showed no pathology in the extracardiac organs. In heart, local and small foci of fibrosis and inflammatory cells were detected with both vectors, congruently with our previous studies^21^.

**Figure 6:**
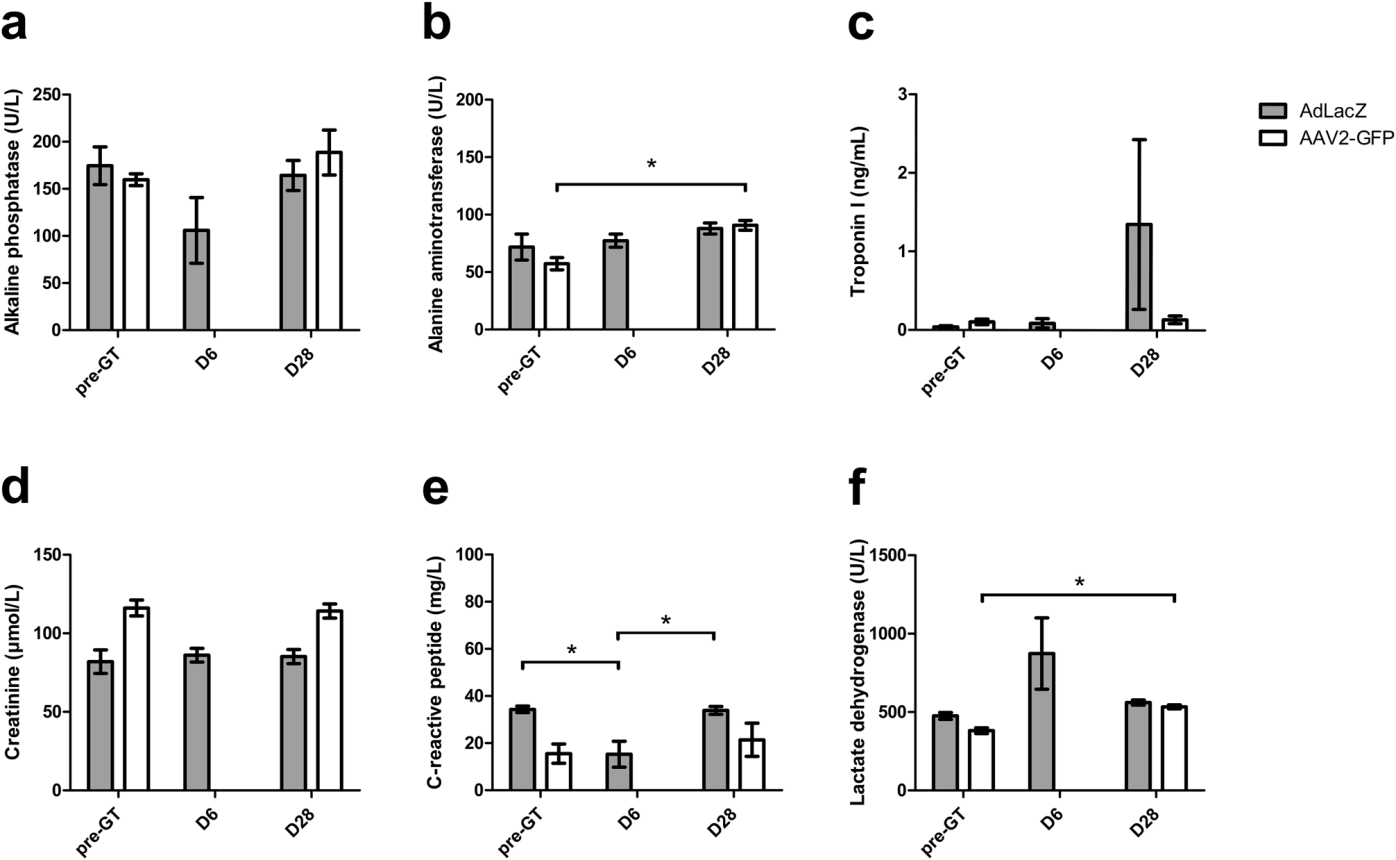
Clinical chemistry. Results are presented as mean ± SEM. * indicates statisticalyy significant (p < 0.05) difference between measurements indicated in square brackets. Grey columns indicate the results of AdLacZ animals. White columns indicate the results of AAV2-GFP animals.

High TnI level was detected in one AdLacZ animal at d28 timepoint, but not at d6 timepoint, which would be the most probable time point to detect operation or gene therapy-related complications^14^. In AAV2 group, statistically significant elevations of ALAT (p = 0.0072, 95% CI = 21.21–45.75) and LDH (p = 0.0201, 95% CI = 57.91–245.9) were detected at d28 timepoint. (Fig. 6).

## Discussion

The ink distribution in the myocardium indicates the maximum distribution potential of this method. However, every viral vector has its own kinetic features that differ from the ink. In this study, we report a wide distribution and transgene expression of Ad and AAV2 vectors in the pig LV anatomically and functionally resembling the human heart. However, the fourth set of experiments showed that even better distribution results could be achieved by adding a transient occlusion of the anastomotic vein to the protocol and using a divided administration protocol for the ACV retroinjection. In this study, we used AAV2 vector, but according to our experience, AAV6 and AAV9 work equally well in pig heart.

The procedure can be performed in a standard cardiological catheterization laboratory and is likely directly applicable to human studies because of the high anatomical and physiological similarity between human and porcine hearts. The LV can be divided roughly into three main blood supply and drainage regions in both species: LVAW and anterior IVS are supplied by LAD and drained via ACV. LVLW is supplied by LCx and drained mainly via LMV and/or LPMV. LVPW and posterior IVS are supplied via LCx and/or RCA and drained by MCV. The vessel pair of LAD and ACV accounts for roughly 50% of the LV circulation. However, the individual anatomy must always be evaluated before performing operational therapies.

By venous approach, the myocardium can be transduced via postcapillary venules, which are highly fenestrated and are not regulated by perivascular sphincters as in precapillary arteries. As the selective pressure-regulated coronary vein infusion ^4^, this method utilizes the advantages of a venous approach without the problems related to prolonged arterial blockage. Moreover, a wider distribution is achieved by three retroinjections to the three main LV veins, instead of infusion into a single vein. Compared to myocardial injections, retrograde injections provide a much broader and more homogenous distribution of viral vectors, in the absence of potential needle injection-related damages.

The procedure requires training, and the relatively long operation time and transient arterial occlusions are obvious safety concerns. Potential complications of the procedure include vascular dissection, myocardial damage, and arrhythmias. However, according to our experience, the risk is relatively low in experienced hands The maximal occlusion time of the arteries was one minute, which is known to be safe and feasible in human percutaneous cardiac interventions. Ultimately, only future studies with larger group sizes will reveal a more accurate approximation of the complication rate and side effects.

The high dose of vectors needed to treat the whole LV increases the risk of transgene expression in the non-target tissues. We showed a very low AAV2 genome presence and non-detectable AAV2-mediated transgene expression in the extracardiac tissues. Instead, Ad seems to be more widely distributed in both the heart and the extracardiac tissues, as transgenic mRNA was also detected outside the heart. Differences in the expression patterns are most likely related to the known differences between the vectors, such as the capsid size and binding profile ^14^. Moreover, transgenic protein-evoked immune reactions can eliminate some transduced cells, affecting the results.

It is concluded that retrograde injections into the three main LV veins are a potential new approach to achieve a global LV gene transfer effect for treating HF and other cardiac diseases.

## Data Availability

All data generated or analyzed during this study are included in this article.
